# A Single-Institution Analysis of 126 Patients Treated with Stereotactic Radiosurgery for Brain Metastases

**DOI:** 10.3389/fonc.2017.00090

**Published:** 2017-05-12

**Authors:** Kevin B. Harris, Melanie R. Corbett, Henry Mascarenhas, Kenneth Stuart Lee, Hyder Arastu, Clinton Leinweber, Andrew W. Ju

**Affiliations:** ^1^Department of Radiation Oncology, East Carolina University Brody School of Medicine, Greenville, NC, USA; ^2^Landauer Medical Physics, Charlotte, NC, USA; ^3^Vidant Neurosurgery, Greenville, NC, USA

**Keywords:** Gamma Knife, radiosurgery, brain neoplasms, stereotactic radiosurgery, underinsured

## Abstract

**Background:**

The objective of this study was to report our institutional experience with Gamma Knife^®^ Radiosurgery (GKRS) in the treatment of patients with brain metastases.

**Methods:**

Retrospectively collected demographic and clinical data on 126 patients with intracranial metastases were reviewed. The patients in our study underwent GKRS at Vidant Medical Center between 2009 and 2014. Kaplan–Meier curves were used to compare survival based on clinical characteristics for univariate analysis, and a Cox proportional hazards model was used for multivariate analysis.

**Results:**

The median age of the patient population was 62 years. Medicare patients constituted 51% of our patient cohort and Medicaid patients 15%. The most common tumor histologies were non-small cell lung cancer (50%), breast cancer (12.7%), and melanoma (11.9%). The median overall survival time for all patients was 5.8 months. Patients with breast cancer had the longest median survival time of 9.15 months, while patients with melanoma had the shortest median survival time of 2.86 months. On univariate analysis, the following factors were predictors for improved overall survival, ECOG score 0 or 1 vs. 2 or greater (17.0 vs. 1.8 months, *p* < 0.001), controlled extracranial disease vs. progressive extracranial disease (17.4 vs. 4.6 months, *p* = 0.0001), recursive partitioning analysis Stage I vs. II–III (18.2 vs. 6.2 months, *p* < 0.007), multiple GKRS treatments (*p* = 0.002), prior brain metastasectomy (*p* = 0.012), and prior chemotherapy (*p* = 0.021). Age, ethnicity, gender, previous external beam radiation therapy, number of brain metastases, and hemorrhagic vs. non-hemorrhagic tumors were not predictors of longer median survival time. Number of metastatic brain lesions of 1–3 vs. ≥4 (*p* = 0.051) and insurance status of Medicare/Medicaid vs. commercial insurance approached significance (13.7 vs. 6.8 months, *p* = 0.08). On multivariate analysis, ECOG performance status 0–1 (*p* < 0.001), multiple GKRS treatments (*p* = 0.003), and control of extracranial disease (*p* = 0.001) remained significant predictors of survival.

**Conclusion:**

ECOG score, control of extracranial disease, and multiple GKRS treatments are predictors of longer median survival following GKRS in our patient population. GKRS is an effective treatment for brain metastases, but these factors may be considered in patient selection for GKRS.

## Introduction

As advances are made in surgical oncology, chemotherapy, and radiotherapy, median survival times for cancer patients continue to improve. Consequently, there are increasing numbers of patients presenting with brain metastases (1). Brain metastases are the most common brain tumor in adults, developing in about 10–40% of adult cancer patients (2). Lung cancer, melanoma, breast cancer, colorectal cancer, and renal cell cancer are the most common sources of brain metastases (3). Stereotactic radiosurgery (SRS) including Gamma Knife^®^ Radiosurgery (GKRS) is one strategy for treating patients with limited metastatic disease to the brain or in patients who have recurrent brain metastasis after prior whole-brain radiotherapy (WBRT). The aims of treatment are palliation of neurological symptoms, maintenance of performance status (PS), and local control of metastatic disease. The benefit of GKRS is reduction of radiation to the surrounding normal brain parenchyma, which may thereby reduce neurological toxicities when compared to WBRT (4, 5) or which may improve local control when used in conjunction with WBRT (6) or surgery (7).

Despite advances in treatment, the prognosis for brain metastases remains poor. Many factors including tumor histology, control of extracranial disease, and PS impact a patient’s overall survival (8). It has been reported that uninsured and underinsured cancer patients often have delayed diagnosis and inferior outcomes (9–11). Our institutional series is unique in that a larger percentage of our patient population is African-American, on Medicaid or on Medicare compared to previous publications reporting health disparities in patients treated for brain metastases (10, 11). The purpose of this study is to examine the impact of demographic and clinical characteristics on overall survival in patients receiving SRS and to compare our outcomes with peer institutions.

## Materials and Methods

### Patient Population

This study reviews the experience of using GKRS in the treatment of brain metastases at the Brody School of Medicine at East Carolina University/Vidant Medical Center. We received University and Medical Center Institutional Review Board (UMCIRB) approval to conduct a retrospective review on the patients treated for metastatic brain lesions using the Gamma Knife (GK) by East Carolina University physicians at Vidant Medical Center between the years 2009 and 2014. A waiver of informed consent was granted by the UMCIRB, which functions to protect the rights and welfare of human subjects in research at our institution. All patients with a treatment diagnosis of metastatic disease to the brain were included in this review. Histologic confirmation of the cancer diagnosis was a requirement for inclusion.

### Stereotactic Radiosurgery

The Leksell Gamma Knife Perfexion (Elekta AB, Stockholm, Sweden) was used for the SRS. Patients were treated on both an outpatient and inpatient basis. All patients were treated with frame-based immobilization and MRI-based treatment planning, with plans developed under the supervision of a radiation oncologist, a medical physicist, and a neurosurgeon. The radiation dose was contingent on tumor diameter/volume, proximity to critical structures, history of prior surgery, and history of prior radiation treatment. Single-fraction SRS was used in all cases in this review. Radiation doses ranged from 10 to 24 Gy with a median dose of 20 Gy. The first follow-up was planned for 4–6 weeks after GKRS, and subsequent follow-up was generally planned at 3-month intervals. GKRS for patients with prior metastasectomy was delivered to the surgical cavity with margin. Patients with any form of insurance (Medicare, Medicaid, or private insurance) were accepted for treatment in our program, some patients with no insurance were also treated, although at our institution many of these patients received SRS using a separate robotic-based system due to the availability of a more flexible payment with that modality.

### Data Collection/Statistical Analysis

The clinical characteristics collected in the study included ECOG PS, control of extracranial disease, recursive partitioning analysis (RPA) class (12), number of GKRS treatments, prior metastasectomy, prior external beam radiotherapy to the brain, prior chemotherapy, concurrent chemotherapy, hemorrhagic metastasis, number of brain metastases, histology, insurance status, gender, age, and ethnicity. Overall survival time was calculated from the time of treatment with first radiosurgery until the date of death from any cause. For surviving patients, the survival time was censored at the date of last follow-up. Survival was estimated by use of the Kaplan–Meier method. Local control and brain control were calculated from the time of initial GKRS within the study period until clear radiographic failure at the treated site and elsewhere outside the SRS field in the brain, respectively. Descriptive statistics were used to describe demographic and clinical characteristics. Univariate analysis was performed using the log-rank test to compare overall survival between patient groups within a factor. A Cox-proportional hazards model was used for multivariate analysis on all clinical factors that were significant or approached significance (*p* < 0.1) in the univariate analysis. All statistical analyses were performed using MedCalc^®^ version 12.6.0.0.

## Results

One hundred and twenty-six patients were identified and included in this review. Demographic and clinical characteristics of the patients are presented in Table 1. The median patient age at treatment was 62 years (range 28–86 years). Non-small cell lung carcinoma (NSCLC) was the most common histologic diagnosis, followed by breast cancer and melanoma.

**Table 1:** **Patient population demographics and clinical characteristics**.

Clinical characteristic	Patient *n* and percentage (total *n* = 126)
Male	61 (48.4)
Female	65 (51.6)
Caucasian	80 (63.5)
African-American	43 (34.1)
Other	3 (2.4)
Medicare	64 (50.8)
Commercial insurance	41 (32.5)
Medicaid	19 (15.1)
Uninsured	2 (1.6)
Single metastasis	48 (38.1)
Multiple metastases	78 (61.9)
Controlled extracranial disease	68 (54.0)
Uncontrolled extracranial disease	58 (46.0)
NSCLC	63 (50.0)
Breast	16 (12.7)
Melanoma	15 (11.9)
Other	32 (24.4)
ECOG 0	36 (28.6)
ECOG 1	53 (42.1)
ECOG 2	37 (29.3)
RPA Stage I	40 (31.7)
RPA Stage II	53 (42.1)
RPA Stage III	33 (26.2)
GPA Score 0.0–1.0	13 (10.3)
GPA Score 1.5–2.0	50 (39.7)
GPA Score 2.5–3.0	47 (37.3)
GPA Score 3.5–4.0	16 (12.7)
1 brain lesion	45 (35.7)
2 or 3 lesions	56 (44.4)
4 or more lesions	25 (19.9)
Multiple GKRS sessions	13 (10.3)
One GKRS session	113 (89.7)
Prior brain surgery	18 (14.3)
No prior brain surgery	108 (85.7)
Median age (range)	62 years (range 28–86)
Median survival (range)	5.8 months (range 0.1–64.1)

*Data are frequency (%) unless otherwise stated*.

*NSCLC, non-small cell lung carcinoma; RPA, recursive partitioning analysis; GKRS, Gamma Knife^®^ Radiosurgery*.

The median overall survival time for the entire cohort was 5.8 months (range 0.1–64.1 months) from initial treatment with GKRS. The results of the univariate analysis of overall survival based on clinical characteristics are described in Table 2. Patients with breast cancer had the longest median survival of 9.15 months, while patients with melanoma had the shortest median survival of 2.86 months. On univariate analysis, predictors for improved overall survival included ECOG PS 0–1 vs. ≥2 (17.0 vs. 1.8 months, *p* < 0.001), controlled vs. uncontrolled extracranial disease (17.4 vs. 4.6 months, *p* < 0.001), RPA Stage I vs. II–III (18.2 vs. 6.2 months, *p* < 0.007), multiple GKRS treatments vs. one GKRS treatment (31.0 vs. 7.0 months, *p* = 0.002), prior brain metastasectomy vs. none (median not reached vs. 7.1 months, *p* = 0.012), and prior chemotherapy vs. none (15.3 vs. 5.2 months, *p* = 0.021). The number of metastatic lesions of 1–3 vs. ≥4 approached significance (15.2 vs. 6.6 months, *p* = 0.051), as did tumor histology of breast cancer vs. all other histologies (*p* = 0.08). Insurance status of Medicare/Medicaid vs. commercial insurance approached significance (13.7 vs. 6.8 months, *p* = 0.08). Previous external beam radiation therapy, concurrent chemotherapy, hemorrhagic metastases, gender, and age were not significant predictors of overall survival. Ethnicity (Caucasian vs. non-Caucasian, *p* = 0.89) was not a statistically significant predictor of improved survival time. Only 74 patients (59%) had postradiosurgery MRIs available for review, of these patients, the median local control is 20.9 months, and the median time of control of the brain outside the SRS field is 13.2 months.

**Table 2:** **Univariate analysis of median overall survival time following GKRS by clinical characteristics**.

Clinical characteristics	Median survival (months)	*p*-Value
ECOG 0 or 1 vs. ≥2	17.0 vs. 1.8	0.0001
Controlled vs. uncontrolled extracranial disease	17.4 vs. 4.6	0.0001
RPA Stage I vs. RPA ≥II	18.2 vs. 6.2	0.0007
Multiple GKRS sessions vs. one GKRS session	31.0 vs. 7.0	0.002
Prior metastasectomy vs. no prior metastasectomy	Median not reached vs. 7.1	0.012
Prior chemotherapy vs. no prior chemotherapy	15.3 vs. 5.2	0.021
Less than 4 metastases vs. ≥4 metastases	15.2 vs. 6.6	0.051
Breast cancer vs. all other histologies	20.4 vs. 8.9	0.08
Commercial insurance or Medicare vs. Medicaid or uninsured	13.7 vs. 6.8	0.08
Female vs. male	15.4 vs. 7.0	0.10
Caucasian vs. non-Caucasian	13.7 vs. 10.1	0.89

*GKRS, Gamma Knife^®^ Radiosurgery*.

A Cox-proportional hazards model was used for multivariate analysis. The model included ECOG PS, control of extracranial disease, RPA class, prior GKRS, prior brain metastasectomy, prior chemotherapy, number of metastatic lesions, tumor histology, and insurance status. On multivariate analysis, ECOG PS 0–1 prior GKRS and control of extracranial disease remained significant predictors of survival, while RPA stage, prior brain metastasectomy, the use of prior chemotherapy, and number of metastatic lesions did not remain significant. Breast cancer vs. other histologies did not remain significant on multivariate analysis (Table 3).

**Table 3:** **Cox proportional hazards model of overall survival by the covariants identified as significant or near-significant on univariate analysis**.

Covariant	*p*-Value
ECOG 0 or 1 vs. ≥2	0.0001
Controlled vs. uncontrolled extracranial disease	0.001
Multiple GKRS sessions vs. one GKRS session	0.003
Prior metastasectomy vs. no prior metastasectomy	0.09
Breast vs. all other histologies	0.11
Less than 4 metastases vs. ≥4 metastases	0.38
RPA Stage I vs. RPA ≥II	0.41
Commercial insurance or Medicare vs. Medicaid or uninsured	0.49
Prior chemotherapy vs. no prior chemotherapy	0.88

*GKRS, Gamma Knife^®^ Radiosurgery; RPA, recursive partitioning analysis*.

## Discussion

We present one of the larger single-institution cohorts of patients treated for brain metastases with GKRS. Our data support previously published reports that GKRS is an effective treatment modality for patients with limited metastatic disease to the brain (8, 13–18). In our study, better ECOG performance statues, control of extracranial disease, and multiple SRS treatments are predictors of improved median overall survival. Ethnicity and insurance status are not predictors of worse outcome following SRS in our patient population.

**Figure 1:**
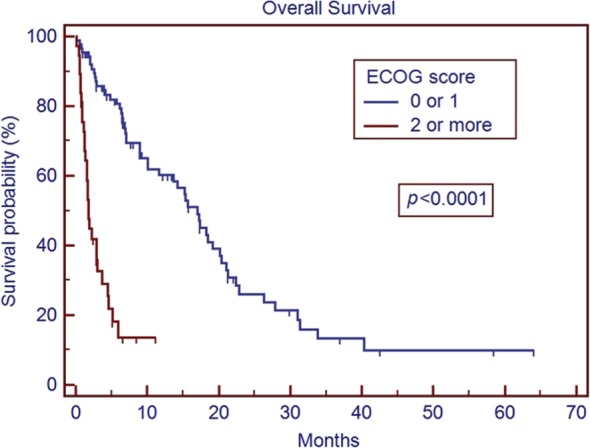
**Overall survival based on ECOG performance status**.

**Figure 2:**
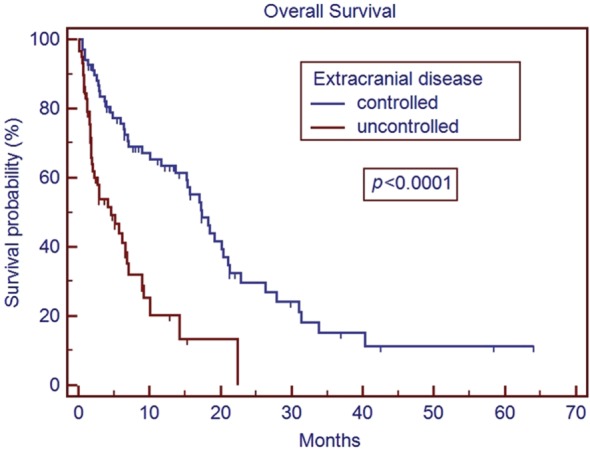
**Overall survival based on extracranial disease**.

In our series, patients with an ECOG PS of ≥2 had a median survival time of 1.8 months (Figure 1). The median survival in patients with ECOG performance status of ≥2 is comparable to the overall survival reported in other studies for patients with untreated metastatic disease (19, 20). While radiosurgery may be used for symptom palliation, the short overall survival in patients with ECOG ≥2 brings to question the additional utility of GKRS in such patients over more conservative management. Our series also matches previous reports that demonstrate improved overall median survival with better control of extracranial disease (21) (Figure 2). This series re-enforces the conclusions that these factors remain strong predictors of survival across different patient populations, including our cohort with many non-Caucasian patients and patients without private insurance.

**Figure 3:**
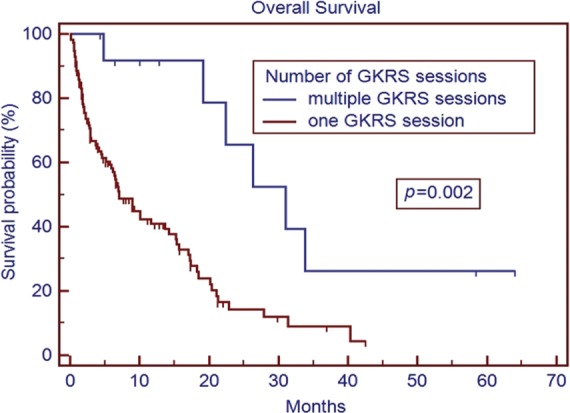
**Overall survival based on number of Gamma Knife^®^ Radiosurgery (GKRS) sessions**.

**Figure 4:**
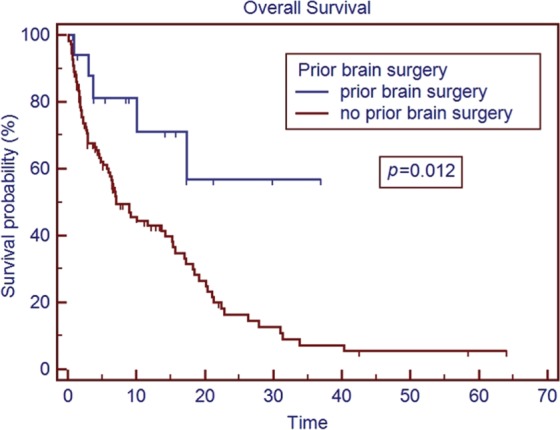
**Overall survival based on prior metastasectomy**.

Patients who were able to receive multiple GKRS sessions had a significantly longer median survival than those treated with a single session (Figure 3). There is a strong selection bias in this result, as patients who are able to receive additional GKRS are not only surviving long enough to have additional follow-up scans but also presenting with a limited number of recurrent CNS lesions to fit our selection criteria for repeat GKRS vs. WBRT or symptomatic care alone. It is difficult to draw strong conclusions from this result at this time, but it does at least appear to support the approach of using repeat GKRS in appropriately selected patients who have regional recurrence of metastatic disease within the brain. Similarly, patients with prior metastasectomy saw a survival benefit on univariate analysis (although not on multivariate analysis) compared to patients with no prior metastasectomy (Figure 4). This may also reflect patient selection as patients who were candidates for metastasectomy generally presented with a single CNS lesion and were clinically regarded as both functioning well enough to be able to better tolerate surgery and also having a long enough expected overall survival to be more likely to benefit from more aggressive local therapy. However, these findings do seem to support the studies showing better outcomes in patients treated with GKRS to the surgical bed (7).

On univariate analysis, RPA Stage I vs. II–III was a significant predictor for improved median survival, but on multivariate analysis, this was no longer significant. This could be related to the fact that the RPA is correlated with ECOG PS and control of extracranial disease by definition, and this could have confounded the multivariate analysis. Prior chemotherapy vs. no prior chemotherapy was another factor that did significantly predict survival on multivariate analysis, this could be due to the heterogeneity of the clinical scenarios pertaining to whether the patient had received prior systemic therapy. The heterogeneity of patients may also affect our analysis of outcome by histology, although we did find that the survival of breast cancer patients vs. all other histologies approached significance, which agrees with other publications showing differences in outcome depending on histology (22, 23). The number of metastatic lesions of 1–3 vs. ≥4 approached significance. There is currently debate in the literature regarding the importance of the number of brain metastases vs. the total tumor volume in predicating overall survival (8, 24, 25). The volume of tumor was not part of this current analysis due to variation in how tumor/GTV volume was documented, but it can be examined more closely in future patients.

**Figure 5:**
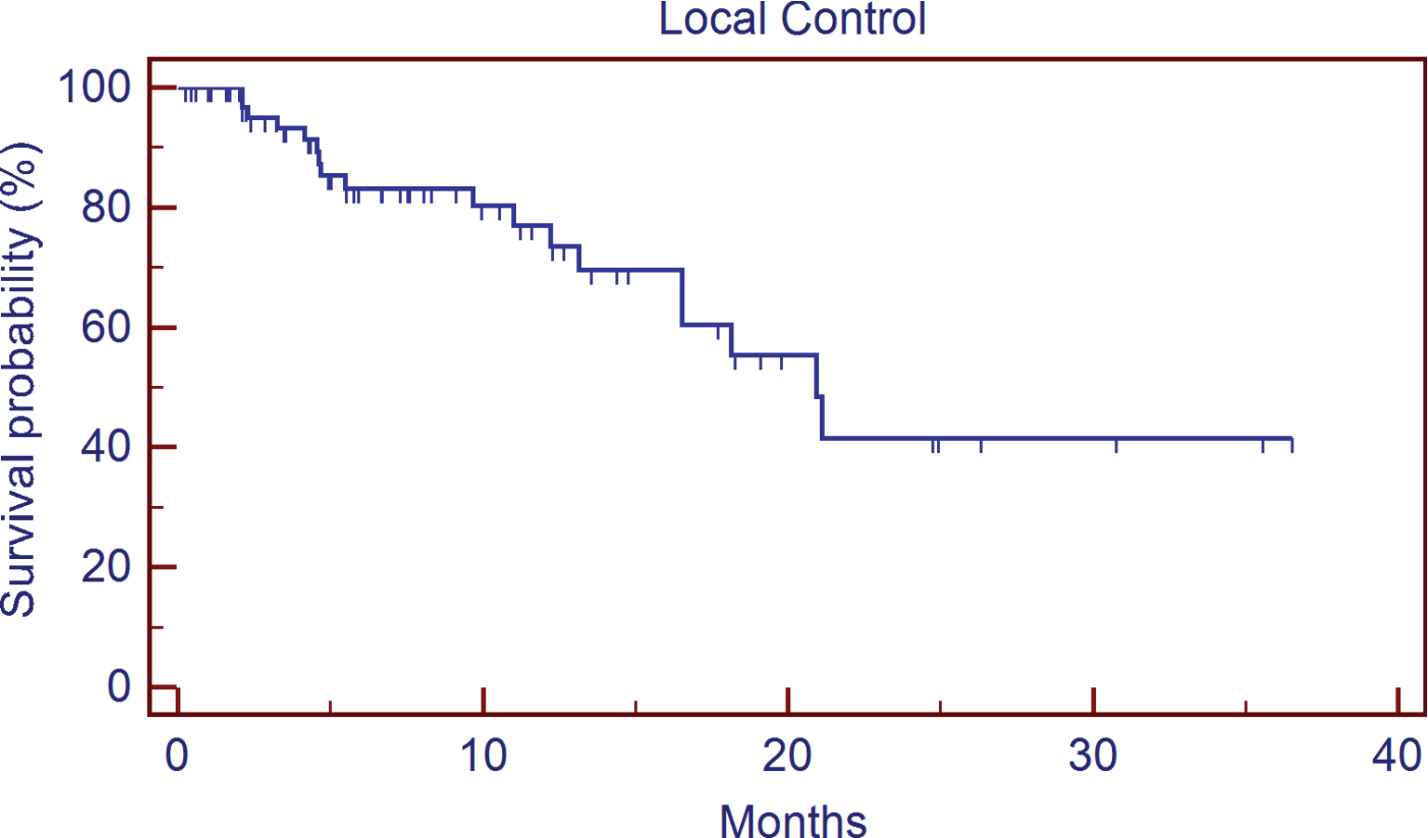
**Local control**.

**Figure 6:**
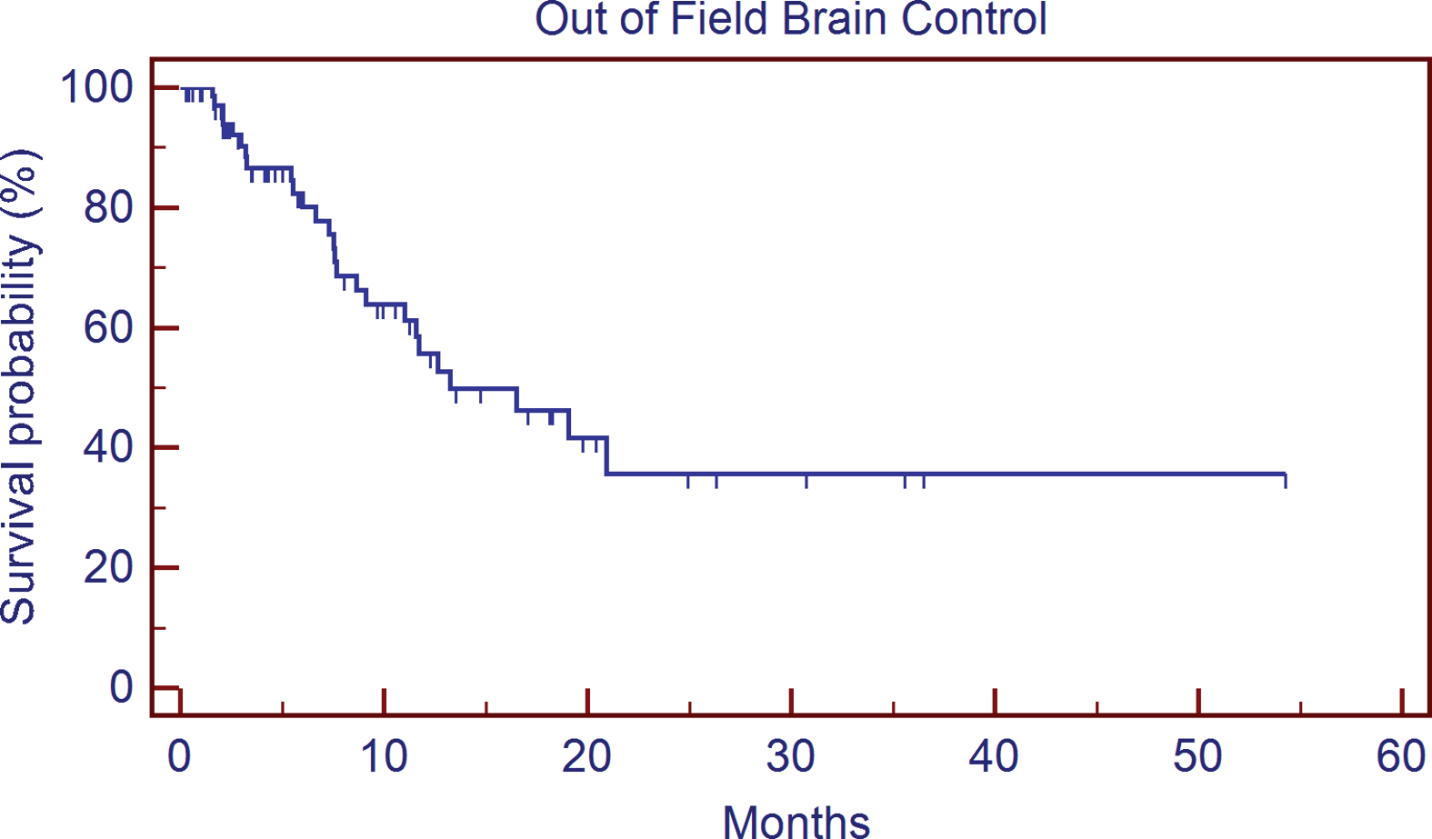
**Out-of-Gamma Knife^®^ Radiosurgery (GKRS) field brain control**.

Ethnicity and gender were also not predictors of worse survival following SRS in our analysis. Previous studies have reported inferior outcomes in cancer patients who are uninsured or underinsured (6). On univariate analysis, patients who were uninsured or with Medicare/Medicaid vs. commercial insurance approached significance (13.7 vs. 6.8 months, *p* = 0.080); however on multivariate analysis, insurance status was no longer a significant predictor of overall survival (*p* < 0.49). This lack of significance could be due in part to the number of patients included in our analysis compared to the numbers of patients in publications using registry data that showed a worse mortality in underinsured patients (10, 11), but it may also suggest that the primary drivers of potential health disparity in our patient population may be related to their presentation in a more advanced disease state (potentially with more active extracranial disease, lower PS, etc.), rather than an inability to receive adequate care following GKRS. One weakness to our study in regard to our analysis of insurance status is the fact that only two uninsured patients were included, as explained in the Section “Materials and Methods” this was mostly due to a more favorable payment schedule available with robotic SRS that is also available at our institution and a preference for most uninsured patients to choose that modality over GKRS. A separate analysis that includes the outcomes of patients treated for brain metastases with robotic SRS, especially uninsured patients, would be interesting but was unfortunately beyond the scope of this particular study. An analysis of local control, brain control, or preservation of neurologic function following GKRS would also have been informative, but unfortunately since our institution treats many patients at considerable geographic distance from our center who preferred to continue follow-up and additional care with their local oncologists, we did not feel as though the quality of the data for those outcomes was thorough enough for inclusion in our analysis. We included the local control (Figure 5) and out of field brain control (Figure 6) curves of the 74 patients with post-GKRS MRIs available for review, our rates of control are roughly equivalent to those seen in other series (23, 25).

## Conclusion

Gamma Knife SRS is an effective palliative treatment for brain metastases in our institutional cohort. ECOG PS 0–1, control of extracranial disease, and multiple SRS treatments are predictors of longer median overall survival following GKRS. Prior brain metastasectomy approached significance in our study. These factors may influence patient selection for SRS. Ethnicity and insurance status are not predictors of worse outcomes following SRS at our institution. Our median survival matches trends seen nationally for patients with advanced disease. Prospective studies to verify that ethnicity and insurance status do not appear to influence the outcomes of patients following GKRS are needed.

## Ethics Statement

This study was carried out in accordance with the recommendations of the guidelines for retrospective reviews of the East Carolina University Institutional Review Board on 12/14/2015, under study number UMCIRB 15-001726. The protocol was approved by the East Carolina University Institutional Review Board.

## Author Contributions

KH: principle author, primary data collection. MC: database manager, submitted IRB, aided in data collection, edited manuscript. HM: aided in patient identification and data collection, edited manuscript. KL: physician who treated patients in this trial, edited manuscript. HA: physician who treated patients in this trial, edited manuscript. CL: physician who treated patients in this trial, edited manuscript. AJ: corresponding author, principle investigator, recipient of grant funding the project.

## Conflict of Interest Statement

The authors declare that the research was conducted in the absence of any commercial or financial relationships that could be construed as a potential conflict of interest.
